# Positron Emission Tomography Techniques to Measure Active Inflammation, Fibrosis and Angiogenesis: Potential for Non-invasive Imaging of Hypertensive Heart Failure

**DOI:** 10.3389/fcvm.2021.719031

**Published:** 2021-08-17

**Authors:** Viktoria Balogh, Mark G. MacAskill, Patrick W. F. Hadoke, Gillian A. Gray, Adriana A. S. Tavares

**Affiliations:** ^1^Centre for Cardiovascular Science, The Queen's Medical Research Institute, The University of Edinburgh, Edinburgh, United Kingdom; ^2^Edinburgh Imaging, The Queen's Medical Research Institute, The University of Edinburgh, Edinburgh, United Kingdom

**Keywords:** PET imaging, hypertensive heart failure, fibrosis, inflammation, angiogenesis

## Abstract

Heart failure, which is responsible for a high number of deaths worldwide, can develop due to chronic hypertension. Heart failure can involve and progress through several different pathways, including: fibrosis, inflammation, and angiogenesis. Early and specific detection of changes in the myocardium during the transition to heart failure can be made via the use of molecular imaging techniques, including positron emission tomography (PET). Traditional cardiovascular PET techniques, such as myocardial perfusion imaging and sympathetic innervation imaging, have been established at the clinical level but are often lacking in pathway and target specificity that is important for assessment of heart failure. Therefore, there is a need to identify new PET imaging markers of inflammation, fibrosis and angiogenesis that could aid diagnosis, staging and treatment of hypertensive heart failure. This review will provide an overview of key mechanisms underlying hypertensive heart failure and will present the latest developments in PET probes for detection of cardiovascular inflammation, fibrosis and angiogenesis. Currently, selective PET probes for detection of angiogenesis remain elusive but promising PET probes for specific targeting of inflammation and fibrosis are rapidly progressing into clinical use.

## Introduction

**Figure 1:**
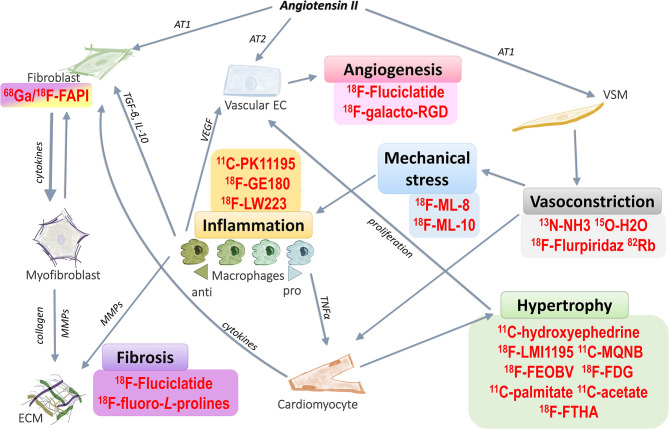
Targeting pathways related to hypertensive heart failure with PET radiotracers. Angiotensin II stimulates a number of different pathways that contribute to cardiac remodeling in hypertensive heart failure. These pathways can be targeted with various PET radiotracers, which can then assess processes related to, but not limited to, hypertrophy, vasoconstriction, mechanical stress, inflammation, fibrosis and angiogenesis. Examples of these radiotracers are shown above. Angiotensin II acting through the AT1 receptor on vascular smooth muscle it can induce vasoconstriction, leading to mechanical stress on the cardiovascular system. As a result, cardiomyocytes enlarge and the heart hypertrophies. Hypertrophy leads to inadequate blood supply to the affected areas in the heart, inducing the proliferation of vascular endothelial cells to form new blood vessels (angiogenesis), and this is enhanced by proangiogenic signals from anti-inflammatory macrophages. Mechanical stress also causes inflammation in the myocardium, enhancing various pro- and anti-inflammatory signaling pathways, which can lead to cardiac remodeling by promoting fibrosis. Angiotensin II also increases proliferation of fibroblasts via the AT_1_ receptor. In contrast, it decreases proliferation of vascular endothelium through activation of the AT_2_ receptor. Fibroblasts (as well as macrophages and vascular endothelial cells) may directly differentiate into myofibroblasts. The main activity of myofibroblasts is the generation of collagen deposits in the ECM (12–15) AT_1_, angiotensin receptor type 1; AT_2_, angiotensin receptor type 2; VEGF, vascular endothelial growth factor; TGF-β, transforming growth factor beta; IL-10, interleukin 10; TNF-α, tumor necrosis factor alpha; MMPs, matrix metalloproteinases; ECM, extracellular matrix. List of PET radiotracers and their application are presented in more detail in Tables 1, 2.

Heart failure is one of the leading causes of death worldwide with ~35% risk of death within the first year after diagnosis (1–3). It is a chronic, debilitating condition which affected around 40 million people globally in 2015 (4). The prevalence of heart failure greatly increases with age and has also increased over the past decades (5, 6). Chronic hypertension has been identified as a cause of heart failure, and is associated with a 2-fold increase in the risk of heart failure in men, and a 3-fold increase in women, compared to those within the healthy range of blood pressure. In addition, lifetime risk of heart failure can double with an increase in blood pressure from under 140/90 mmHg to over 160/100 mmHg (7). Angiotensin II, which has an important role in the development of hypertension and associated cardiovascular and hypertensive heart disease (8–11), influences a number of signaling pathways implicated in the pathogenesis of heart failure (Figure 1). Angiotensin-II-induced inflammation and fibrosis in the myocardium, due to increased pressure overload, contributes to the development of heart failure (11). It has also been suggested that angiotensin II treatment in mice can give rise to fibroblast populations in the heart which are unrelated to myofibroblasts (16).

Imaging techniques can help assess the function and morphology of the myocardium, both in the healthy heart and during the development and progression of heart failure. Several imaging modalities are available in the clinical setting, including ultrasound, computerized tomography (CT), magnetic resonance imaging (MRI), and molecular imaging techniques [single photon emission computed tomography (SPECT), and positron emission tomography (PET)] (17). PET imaging provides quantitative information on biological processes in the living body by quantifying the distribution and uptake of a radiotracer. This technique has high sensitivity for detection of molecular changes and is uniquely placed to investigate disease activity *in vivo* in the context of heart failure. The use of radiotracers allows for high signal specificity by exclusively targeting distinct cellular processes at the molecular level, thus allowing for the detection of changes at early time points during pathology (18).

The most established cardiovascular PET radiotracers target myocardial perfusion and viability; however, there is a wide range of pathways and processes which can be imaged using these radiotracers, either pre-clinically or in the clinic. Table 1 summarizes the properties of key radiotracers used in classical cardiac PET imaging, with examples and further details of radiotracers targeting perfusion, metabolism, viability, cell death innervation. Table 2 lists current and emerging radiotracers used for imaging angiogenesis, extracellular matrix (ECM) remodeling and inflammation.

**Table 1:** Classic PET radiotracers for cardiac imaging applications.

**Application**	**PET radiotracers**	**Clinical/preclinical use**
Myocardial perfusion	^82^Rb	Coronary flow reserve/stenosis assessment, decreased uptake associated with disease (19)
	^13^N-NH_3_	Assessment of stenosis and coronary artery disease. Radiotracer uptake is decreased in areas of ischemia in the myocardium (20)
	^15^O-H_2_O	Assessment of regional myocardial blood flow in cardiomyopathies which decreased in ischemic areas (21)
	^18^F-Flurpiridaz	Assessment of myocardial perfusion defects, more apparent reduction in uptake with disease than SPECT with ^99m^Tc-sestamibi (22)
	^18^F-BMS-747158-02	High myocardial uptake in rat, rabbit and non-human primate models; perfusion deficit clearly identifiable in rats with permanent left coronary ligation or reperfusion (23)
Metabolism and viability	^18^F-FDG	Application based on glucose metabolism and glucose/radiotracer uptake into tissues: reduced uptake in severely ischemic myocardium with decreased viability while still viable areas with only mild ischemia can exhibit increased uptake. In heart failure, uptake was found to be decreased. (24)
	^11^C-glucose	Similar application to ^18^F-FDG, showed more accurate measurements of regional myocardial glucose utilization rate in dogs (25)
	^11^C-palmitate	Assessment of myocardial metabolism in idiopathic dilated cardiomyopathy (IDCM). ^11^C-palmitate as a measure of fatty acid metabolism which decreases in IDCM (26)
	^18^F-Fluoro-6-Thia- Heptadecanoic Acid	Assessment of fatty acid uptake, in patients with congestive heart failure radiotracer uptake was increased in the myocardium (27)
	^18^F-FTP	Assessment of fatty acid oxidation in *ex vivo* rat heart; data showed a decrease in the hypoxic myocardium (28)
	^18^F-FCPHA	Shown to have potential for studying myocardial fatty acid metabolism preclinically, currently assessed for use in coronary artery disease in Phase II trials (29)
	^18^F-FTO	Analog of 4-thia oleate, assessment of fatty acid oxidation investigated in naïve rats showed promising results for the uptake of the metabolically active radiotracer into the myocardium (30)
	^18^F7*	Assessment of myocardial long chain fatty acid metabolism in rats; showed high uptake into the myocardium in naïve rats, thus a promising target in disease models (31) *15-(4-(2-[^18^F]fluoroethoxy)phenyl)pentadecanoic acid
	^11^C-methionine	Assessment of amino acid metabolism and protein synthesis. In patients with myocardial infarction, it showed increased uptake in the infarct areas (32)
	^13^N-glutamate	Assessment of myocardial ischemia in patients with coronary artery disease showed this radiotracer was more suited for quantification of flow rather than metabolism in humans (33)
Cell death	^18^F-ML-10, ^18^F-ML-8	Quantification of cardiomyocyte apoptosis in rats after myocardial infarction, high radiotracer uptake shown in the infarct area (34)
Sympathetic and Para-sympathetic innervation	^11^C-hydroxyephedrine	Assessment of regional abnormalities in cardiac sympathetic innervation; reduced retention of the radiotracer in chronic heart failure associated with worse outcomes (35)
	^11^C-CGP12177	Quantification of β-adrenergic receptor density in patients with idiopathic dilated cardiomyopathy, where receptor density is decreased (36)
	^11^C-CGP12388	Quantification of β-adrenergic receptor density in patients with idiopathic dilated cardiomyopathy showed reduced density with disease (37)
	^18^F-LMI1195	Quantification of cardiac nerve terminals for assessment of changes in cardiac sympathetic function in a heart failure rat model. Radiotracer uptake in the myocardium was decreased with progression of heart failure (38)
	^11^C-MQNB	Assessment of muscarinic receptors in the heart in chronic heart failure, increased density of receptors measured with the radiotracer (39)
	^18^F-FEOBV	Binds the vesicular acetylcholine transporter; promising results in healthy subjects for the evaluation of parasympathetic innervation in the myocardium (40)

**Table 2:** Current and emerging PET radiotracers for cardiac imaging of angiogenesis, extracellular matrix remodeling, the renin-angiotensin system and myocardial inflammation.

**Application**	**PET radiotracers**	**Clinical/preclinical use**
Angiogenesis	^18^F-Fluciclatide	α_v_β_3_ integrin-selective radiotracer to investigate myocardial repair following infarction, uptake was increased in infarcted regions with better repair, and predicted areas of recovery in patients (41)
	^18^F-galacto-RGD, ^68^Ga-NODAGA-RGD, ^68^Ga-TRAP(RGD)_3_	All used for α_v_β_3_ integrin imaging, to monitor angiogenic repair mechanisms after myocardial infarction in rats. Uptake of ^68^Ga-labeled radiotracers was comparably increased to ^18^F-galacto-RGD in the infarct area (42)
	^64^Cu-NOTA-TRC105	Assessment of newly formed blood vessels in a myocardial infarction rat model to investigate ischemia-induced angiogenesis, with increased uptake in the infarct zone at earlier time-points (43)
ECM remodeling	^68^Ga-FAPI-04	Assessment of fibroblast activity after myocardial infarction in rats with a radiolabeled fibroblast activation protein inhibitor. Uptake increased in the border areas of the infarcted myocardium (44)
	^18^F-FXIII	Assessment of extracellular matrix crosslinking after myocardial infarction in mice. Radiotracer uptake was increased in the heart after infarction (45)
	^18^F-fluoro-*L*-prolines	Assessment of myocardial fibrosis in a myocardial infarct rat model. Uptake was increased in the infarct area with *trans* and in the remote myocardium with *cis* isomer (46)
Renin-angiotensin system	^11^C-KR3117	Targets the angiotensin receptor type 1, was shown to have increased uptake in the infarct area in pigs after myocardial infarction compared to the remote areas. Also shown to be safe to use in humans (47)
Myocardial inflammation	^18^F-FDG	Assessment of inflammation in myocardial infarction. Uptake is associated with the increase in macrophages around infarct region, but signal can be obscured by the radiotracer's metabolic properties (48)
	^18^F-Fluoromethyl-PBR28, ^18^F-CB251	Both bind TSPO (18 kDa translocator protein), a marker of inflammation; used in for the assessment of experimental autoimmune myocarditis in rats. ^18^F-CB251 showed more specific uptake, corresponding to TSPO-rich areas (49)
	^68^Ga-pentixafor	Targets the chemokine receptor CXCR4, increased uptake after myocardial infarction in mice coinciding with upregulation of inflammatory cells. Patient-data more variable (50)
	^11^C-methionine	Based on the accumulation of methionine in macrophages, uptake was most pronounced in inflammatory macrophages and was increased in myocardial infarct areas at 3-day post-injury in mice (51)
	^18^F-GE180	Targets TSPO, showed increased uptake after myocardial infarction in mice at the infarct site at 1-week post-injury and in remote areas during heart failure progression 8 weeks post-injury. Similar results in patients after myocardial infarction (52)
	^18^F-LW223	Targets TSPO, showed increased uptake in infarct areas 7 days following myocardial infarction in rats, consistent with results from macrophage immunostaining (CD68, TSPO). Not susceptible to the rs6971 genetic polymorphism (53)

A critically important clinical question is how we can assess the extent of molecular changes, such as fibrosis, inflammation and angiogenesis, in the heart during development and progression of heart failure. Targeting markers of active disease would provide a more accurate representation of each individual's condition, enabling delivery of more personalized medical care. This specific-targeting PET approach could help differentiate those in urgent need of interventions, e.g., with ongoing active or early stage fibrosis, from those who had an older injury that have established non-active tissue scars. Importantly, cardiac molecular imaging for heart failure diagnosis would be beneficial for determining prognosis and adequate interventions and treatments (54, 55). Despite the well-established treatments and interventions available for hypertension, a significant majority of those who have the condition do not have it under control, according to a US based assessment, only 43.5% of those who have hypertension have it under control. Even though, this shows great improvement since the 2000s, the overall burden of hypertension increased consistently by over 21 million by 2016 despite a 3% decrease in prevalence (56). There is also opportunity for investigating whether the damage done to the myocardium prior to patients being diagnosed could be reversed. PET radiotracers could be used to assess pharmacological interventions.

The variety of pathways and radiotracers included in this review indicates that PET radiotracers explored for one type of pathology have the potential to become valuable tools in other conditions where the same pathways are involved. This review will discuss established and emerging PET techniques that may be useful in assessing conditions such as hypertensive heart failure.

## Classic PET Imaging Techniques for Assessment of Basic Cardiac Physiology and Pathophysiology

### Perfusion, Metabolism and Viability

In the clinic, the main application of imaging with radiotracers in heart failure is the assessment of myocardial perfusion to determine viability and potential ischemia, often via SPECT imaging (57). PET perfusion radiotracers previously used for cardiovascular imaging of perfusion include ^82^Rb, ^13^N-NH_3_, ^13^O-H_2_O and ^18^F-Fluripiridaz (Table 1) (23, 58, 59). In addition to perfusion measurements, most cardiovascular PET imaging studies focus on assessing myocardial metabolism and viability using ^18^F-FDG, a marker of glucose metabolism that has over 90% accuracy for prediction of future recovery of myocardial function (60, 61). However, because ^18^F-FDG is a glucose analog, it is also taken up from the circulation by cells (e.g., macrophages) which are metabolically active as a result of active inflammation (62); thereby confounding assessment of myocardial viability. Unsurprisingly, due to its association with inflammatory cells, ^18^F-FDG has been used to investigate myocardial and coronary inflammation in several diseases, including myocardial infarction and sarcoidosis (63–65). Although widely disseminated in the clinical arena, the lack of specificity associated with ^18^F-FDG can make it difficult to distinguish between myocardial viability and tissue inflammation. Furthermore, ^18^F-FDG has high uptake in healthy myocardium that can mask areas with lower uptake, skewing the results of image analysis (66).

### Cell Death

Cardiac remodeling as a component of heart failure has been associated with increased rates of cell death in the myocardium, with the activation of related signaling pathways due to ischemia and pressure overload (67, 68). Radiotracers for investigating cell death include ^18^F-ML-8 [^18^F-labeled 2-(3-fluoropropyl)-2-methyl-malonic acid] and ^18^F-ML-10 [^18^F-labeled 2-(5-fluoropentyl)-2-methyl-malonic acid] (Table 1) (34). These small molecule compounds are part of the ApoSense family, and they recognize the membrane phospholipid scrambling of apoptotic cells where they then accumulate (69). These have been applied in the setting of myocardial infarction (permanent left coronary artery ligation) in rats to investigate apoptosis in the forming scar and thus disease progression (34). In this study, both ^18^F-ML-8 and ^18^F-ML-10 were used in combination with cardiac ultrasound and PET imaging with ^18^F-FDG to visualize metabolically-active myocardium. The PET signal of both radiotracers targeting apoptosis was high in the areas where ^18^F-FDG showed no uptake, suggesting the cells labeled did not have active metabolism which is indicative of infarct regions (34).

### Cardiac Innervation

Changes in signals from the autonomic nervous system can affect progression of heart failure, with an associated increase in sympathetic drive shown to be worsening the condition, whereas parasympathetic activity has been suggested to be cardioprotective (70, 71). The integrity of both can be investigated with PET radiotracers. ^11^C-hydroxyephedrine [(*N-methyl*-^11^C)-metahydroxyephedrine or ^11^C–*m*HED] is the most commonly used PET radiotracer for imaging cardiac sympathetic innervation (Table 1). It has been used to visualize sympathetic nerves in the heart, focusing on the reuptake of norepinephrine at nerve terminals, and this could be a useful way to investigate the increased sympathetic drive in heart failure (72). Another radiotracer that targets sympathetic innervation is ^18^F-LMI1195 {N-[3-bromo-4-(3-18F-fluoro-propoxy)-benzyl]-guanidine}, which provides an option with a longer half-life compared to ^11^C- *m*HED, while investigating the same area of physiology. It targets the noradrenaline transporter, and in a rabbit model of regional cardiac sympathetic denervation, it successfully mapped sympathetic denervation in the myocardium (73). It should be noted, however, that this radiotracer when used in a mouse model of myocardial infarction was not successful at mapping presynaptic norepinephrine transporters, important for assessing sympathetic function, as opposed to imaging with ^11^C–*m*HED (74). Radiotracers for imaging the parasympathetic system with PET include ^11^C-methylquinuclidinyl benzilate (^11^C-MQNB) and ^18^F-fluoroethoxybenzovesamicol (^18^F-FEOBV); although this type of imaging is associated with physiological limitations due to the myocardium having a low density of cholinergic neurons (75, 76). ^11^C-MQNB, a muscarinic antagonist, was used to target and investigate active muscarinic acetylcholine receptors in the heart and suggested that the highest accumulation of these receptors was in the ventricular septum. It was also suggested that a conformational change of the muscarinic receptor could increase the affinity for the radiotracer, thus the physiologically active receptors were able to bind the radiotracer more readily (75). The radiotracer ^18^F-FEOBV binds to the vesicular acetylcholine transporter of cholinergic neurons and the reduction of this transporter can initiate cardiac remodeling and heart failure (77). Experiments carried out *in vitro* suggested that this tracer would have limited translatability to *in vivo* cardiac studies due to low radiotracer retention and low density of cholinergic neurons in the heart (75, 76). ^11^C-CGP12177 PET was used to assess myocardial β-adrenergic receptor density in patients with non-ischemic cardiomyopathy to investigate left ventricular dysfunction where cardiac sympathetic regulation is affected (78). This study found that the receptor density was lower in patients and showed a significant difference in the severity of heart failure, meaning that those with severe heart failure classification had lower density of β-adrenergic receptors. Another radiotracer, (*S*)-[^11^C] CGP12388, has been successful in detecting a reduction (compared with controls) in myocardial β-adrenergic receptor density in patients with idiopathic dilated cardiomyopathy (37). Therefore, assessment of sympathetic innervation with PET in heart failure can also be valuable in classifying disease severity.

## Emerging PET techniques for quantification of processes involved in the pathogenesis of cardiac remodeling

### Renin–Angiotensin System Imaging

Radiotracers exploring myocardial remodeling through this pathway include those targeting angiotensin II type 1 (AT1) receptors and angiotensin-converting enzyme-1 (ACE-1) (47, 79, 80). [^11^C]-KR31173, a radiotracer targeting AT1 receptors showed an upregulation of the signal in the infarct area (compared with remote areas of the myocardium) in pigs with myocardial infarction. It was also shown to be safe to use in healthy human volunteers (47). Another AT1 antagonist radiotracer, (^18^F)FV45, derived from valsartan had promising results when assessed for visualizing AT1 receptor distribution in rats and was shown to be selective as its uptake was successfully blocked by valsartan pre-treatment (79). A SPECT radiotracer, Tc-Lis (technetium-99m–labeled lisinopril/an ACE inhibitor drug) successfully detected upregulation of ACE-1 in transgenic rats which overexpress this enzyme (80). This suggests it has potential as a tool for monitoring ACE-1 upregulation in heart failure in patients.

### Fibrosis

There are two main types of fibrosis in the injured heart: reactive and replacement fibrosis. Reactive fibrosis (interstitial fibrosis) happens further from the place of the injury as a response to pathological changes in the tissue elsewhere (e.g., infarct area) or as a response to changes in the physical or chemical environment (e.g., pressure overload or hypertension; myocardial inflammation). This can lead to stiffness of the ventricle wall and, thus, increased risk of heart failure. Replacement fibrosis occurs when excess collagen is deposited and fibroblasts replace the ischaemic (or necrotic) tissue at the injury site after myocardial infarct and form a scar to prevent rupturing of the wall due to the loss of the original heart muscle cells (12, 81).

Fibroblast activation is essential for the development of fibrosis in the heart. During cardiac remodeling, myofibroblasts produce collagen, cause interstitial fibrosis and increase collagen deposition. Cardiac remodeling, either to compensate for the loss of myocardial tissue (e.g., in myocardial infarction) or to allow the heart to adapt to the changed environment (e.g., hypertension) can lead to heart failure via development and progression of tissue fibrosis (12). Both *in vitro* and *in vivo*, the renin-angiotensin-aldosterone system, and especially angiotensin-II, have been associated with increased fibrosis and collagen synthesis (82–84). Currently, techniques available for direct imaging of cellular and molecular composition of active cardiac fibrosis are limited (12). There are also no radiotracers for imaging active fibrosis in the routine clinical setting. This type of tracer would greatly enhance diagnostics and management of patients with heart failure, although active clinical research studies are rapidly generating promising results with newly developed PET radiotracers targeting fibrosis.

Several processes during the initiation of fibrosis have been, or could be, targeted with novel and emerging PET radiotracers. These include: immune activation; leaking of the vasculature and coagulation; fibroblast recruitment and proliferation; activation of fibroblasts and myofibroblast differentiation; as well as the resulting ECM crosslinking and the accumulation of matrix components (85). Radiotracers used to investigate another aspect of tissue remodeling, fibroblast activation in cancers, are ^68^Ga-FAPI-2 and ^68^Ga-FAPI-4. The latter accumulated at the injury border after myocardial infarction in rats, making it a potential candidate for assessing fibroblast activation in the context of myocardial remodeling (Table 2) (44, 86).

Integrins play an important role in the adhesion of ECM components to the cellular parts of the tissue; thus radiotracers targeting integrin expression have been identified as good candidates for monitoring progression of fibrosis (87). A large group of radiotracers that were previously explored as markers of integrin expression are those targeting integrins α_v_β_3_ (Table 2). An example of an integrin radiotracer for imaging fibrosis and angiogenesis is ^18^F-Fluciclatide, which targets the α_v_β_3_ integrin and was shown to have increased uptake at areas of recent injury after myocardial infarction in patients (41). Further integrin targeting radiotracers have been assessed in applications other than for myocardial imaging. Integrin α_v_β_6_, an activator of TGF-β, is upregulated during tissue injury on epithelial cells, while increased expression has been demonstrated in fibrosis (88–90).

Other more direct options for targeting the ECM components which accumulate in the myocardium during fibrosis, instead of focusing on the wider pathway processes with integrins, include imaging with radiotracers for elastin or collagen, which are more specific indicators of fibrosis (91). Imaging elastin content, as opposed to collagen, could produce a higher background signal due to the comparably higher levels of elastin in the healthy heart. In addition, elastin appears to accumulate later than collagen in the disease process, thus collagen increases could be detected earlier during remodeling. One example of an elastin radiotracer is ^18^F-AlF-NOTA-EBM which was tested for targeting atherosclerotic plaques, but was not successful at differentiating between plaques and controls (92). To investigate infarct healing after MI and the involvement of inflammation, the tissue transglutaminases radiotracer ^18^F-FXIII (transglutaminase factor-XVIII) was used to visualize the infarct area and matrix crosslinking in the mouse heart (45).

Targeting collagen biosynthesis itself can also be a good strategy to quantify fibrotic activity, by observing the levels of active collagen accumulation. PET imaging can target collagen biosynthesis by the incorporation of radioactive ^18^F-fluoro-proline isomers into actively forming collagen. There are, however, four different isomers of fluoro-proline: *cis*-*L, cis*-*D, trans*-*L* and *trans*-*D*, all of which are safe to use in mice, rats, rabbits, and humans (93). The *D* isomers are less well-characterized and less stable than the *L* isomers, and they also possess lower affinity. Consequently, they are not optimal for studies investigating active collagen biosynthesis (94). Therefore, the *L* isomers are the preferred probes for mapping collagen biosynthesis *in vivo*. Biomarkers for quantifying collagen biosynthesis in the heart via radioactive tagging of fluoro-proline are particularly promising for two main reasons. Firstly, proline and hydroxyl-proline contribute almost a quarter of the amino acids in collagen (95). Secondly, proline is found almost exclusively in collagen. The use of ^18^F-fluoro-proline radiotracers to target active fibrosis in heart failure also has a great potential to be clinically translatable for detection of active fibrosis, as they are present in the precursors of collagen. This is advantageous over other radiotracers which only visualize established fibrotic tissue through directly measuring the end-product (e.g., ECM) of the related pathways such as collagen or elastin; or radiotracers targeting activation of fibroblasts [e.g., fibroblast activation protein (FAP) specific radiotracers] that are known to label active fibrosis and chronic reactive inflammation in the oncology setting (96, 97) and might be also labeling both processes in the cardiovascular context. This is because fibroblast activation occurs following pro-inflammatory Damage-Associated Molecular Patterns (DAMPs) released by dying cardiac cells and activated myofibroblasts produce structural extracellular matrix proteins and matricellular macromolecules (98). Moreover, the ability to image with both *cis-* and *trans-*fluoro-prolines is useful for determining the content of degraded or to-be-degraded immature collagen (in reactive fibrosis) and triple helix collagen (scar tissue), respectively. The *cis-*fluoro-proline isomer has been assessed in a rabbit lung fibrosis model, which showed promising results in the radiotracer's ability to identify fibrosis *in vivo* (99). Currently preclinical cardiovascular studies with ^18^F-fluoro-prolines are underway to determine their utility in the context of myocardial infarction and hypertensive heart failure (46).

### Inflammation

Inflammation and fibrosis are distinct yet interconnected processes, as unresolved inflammation causes fibrosis and fibrosis may lead to chronic inflammation; both are essential for tissue repair after injury (100). Importantly, chronic inflammation and progressive fibrosis lead to increased tissue breakdown and functional impairment of the heart (101–103). Proteins and various components of the ECM influence the inflammatory cascade directly or by acting on signaling pathways triggered by localized insults and also systemic inflammation (100, 104). Macrophages are immune cells which can interact with fibroblasts and directly promote fibrosis and, thus, ECM deposition (105–107). In the angiotensin-II-induced hypertension model, which leads to hypertension via volume and pressure overload on the cardiovascular system, blood derived Ly6C^high^ monocytes were recruited and gave rise to all cardiac macrophage populations in mice (108). Angiotensin II infusion in mice also led to proliferation of residential macrophages in the myocardium, thus during cardiac inflammatory processes, the local macrophage pool expands through both local proliferation and recruitment of blood monocytes (108). Macrophages are also involved in secreting ECM components and are especially important sources of matrix metalloproteinases (MMPs) and tissue inhibitor of metalloproteinases (TIMPs) which can be used as biomarkers of heart failure (109, 110). On the anti-inflammatory side of the spectrum, macrophages have also been shown to degrade collagen in the ECM (111). Recently, resident cardiac anti-inflammatory macrophages were shown to be key determinants in the development of angiotensin-II mediated myocardial fibrosis (112).

PET imaging can selectively identify active inflammation by targeting important proteins expressed in inflammatory cells (113). It is therefore possible to image activated macrophages indicative of inflammation (Table 2). Inflammation after myocardial infarction can be imaged with ^18^F-FDG and ^11^C-methionine ligands, although these radiotracers do not provide a selective or direct measurement of inflammation, as they measure glucose metabolism and cell proliferation, respectively (51, 114, 115). It is notable, however, that uptake of ^11^C-methionine was high after myocardial infarction in the infarct area, where it showed macrophage uptake but not taken up by the impaired cardiomyocytes, indicative of inflammation in the area and thus could provide a more specific assessment than ^18^F-FDG (51). Inflammation in atherosclerotic plaques has also been assessed with radiotracers, such as ^68^Ga-DOTATATE, targeting the somatostatin receptor. ^68^Ga-DOTATATE uptake was increased in lesions with high macrophage content (116). The radiotracer ^68^Ga-DOTANOC, which targets the somatostatin receptor, provided promising results for diagnosis of cardiac sarcoidosis in patients (117). Chemokine receptors can also be targeted in relation to inflammation. One example is CXCR4 which can be targeted by ^68^Ga-pentixafor; uptake of this tracer was increased after myocardial infarction. This uptake coincided with the infiltration of inflammatory cells but did not distinguish between uptake by pro- and anti-inflammatory macrophages (50). Another chemokine radiotracer is ^68^Ga-DOTA-ECL1i which targets the CCR2 (C-C chemokine receptor type 2) and was found to be localized to tissue injury sites in mouse models of cardiomyocyte ablation and myocardial infarction (118). ^64^Cu-DOTA-ECL1i was also investigated to target CCR2+ cells in heart injury mouse models, where it was comparable to the ^68^Ga-labeled probe (119). ^18^F-FDM (^18^F-labeled mannose) has also been used to image inflammation of atherosclerotic plaques in rabbits, where it showed increased uptake within the aortic plaques. This tracer has potential for targeting the mannose receptors on alternatively activated macrophages within the plaques as well as in other areas (120).

The18kDa translocator protein (TSPO), which is present in the outer membrane of the mitochondria of macrophages (121, 122), has been proposed as another target for imaging cardiac inflammation. TSPO was shown to be present both in classically- and in non-classically-activated macrophages. However, there were differences in radiotracer uptake between the types of macrophages, such as in the case of ^18^F-GE180, which shows a binding preference for pro-inflammatory macrophages (113, 123, 124). Understanding the underlying inflammatory and anti-inflammatory mechanisms involved in cardiovascular diseases could be achieved via molecular imaging of TSPO. Therefore, TSPO is likely to be a valuable target to investigate in the setting of myocardial inflammation in heart failure. TSPO has been shown to target macrophage driven cardiac inflammation more selectively than glucose metabolism tracing (124). Although TSPO is expressed in the healthy heart, a radiotracer with high selectivity toward TSPO with concomitant low non-specific binding would enable detection of subtle changes in TSPO expression even without a zero background owing to the high sensitivity of PET for detecting changes in the pM to nM range. Notwithstanding isolation of the inflammatory cell contribution to the measured PET signal could be challenging due to the mixed substrate in hypertensive heart failure where TSPO expression might be increased in cardiomyocytes.

Previously, several ligands have been developed for targeting TSPO with PET. One of these, ^11^C-PK11195, has a low signal-to-noise ratio and high non-specific binding; whereas an alternative ligand, ^11^C-DAA1106, demonstrated superior binding to TSPO, compared with ^11^C-PK11195, when investigating microglia and inflammation in the brain (125–129). TSPO-targeted imaging with the PET radiotracer ^18^F-GE180 also detected the increased inflammation after myocardial infarction both in mice and in humans (52). TSPO radiotracers can have a number of limitations, including: low signal-to-noise ratio; non-specific binding; and perhaps most importantly, alterations in tracer binding as a result of variations in the gene encoding TSPO in humans (125–130). The affinity of several radiotracers for TSPO is affected by the rs6971 human polymorphism, which means that the binding affinity of some can vary depending on which variant of the polymorphism is present. Thus a patient's genetic information would need to be assessed for full analysis of scans acquired with these ligands (130). A new selective PET radiotracer for imaging TSPO, ^18^F-LW223, has the potential to overcome this issue. ^18^F-LW223 has been shown to reversibly bind to its target, can be displaced by PK11195 (which indicates specificity to TSPO) while it also showed uptake consistent with macrophage infiltration after myocardial infarction in rats (53). It is potentially more readily clinically-translatable due to having similar affinity to all TSPO isoforms regardless of the presence of the previously described rs6971 human polymorphism (53, 130). Further examples of TSPO radiotracers with low sensitivity to this polymorphism are ^18^F-FEBMP (*R*)-^18^F-NEBIFQUINIDE and (*R, S*)-^18^F-GE387 (131–133). Another TSPO radiotracer, ^18^F-FEDAC, has been used to monitor liver fibrosis in rats, as the signal correlated well with expression of TSPO in macrophages, which was increased with disease (134). This is promising in relation to using PET to investigate inflammation-induced fibrosis and TSPO expression in macrophages in the failing heart.

### Angiogenesis

Reduced blood supply to the heart tissue through impaired angiogenesis and neovascularization damages the myocardium during heart failure, and can lead to cell death due to oxidative stress and enhanced fibrotic response after cell loss (135). During hypertension, excess mechanical demand on the heart can induce hypertrophy of the myocardium leading to reduced blood supply in the newly enlarged areas resulting in hypoxia and tissue impairment and eventually heart failure. Capillary angiogenesis is important to restore function to these areas. The imbalance of capillary numbers in the heart is a consequence of myocardial enlargement, causes hypoxia in the tissue, and can lead to heart failure (136–138). Improving this capillary imbalance with pro-angiogenic mediators can improve functional outcome during heart failure. For-example neonatal rabbits undergoing aortic banding (and therefore developing pressure overload and hypertrophy) had improved angiogenesis when treated with VEGF while matrix metalloproteinase activity also increased (139). This suggests that angiogenesis and angiogenic factors can have an important role in extracellular and vascular remodeling in the myocardium. Pro-angiogenic stimuli can lead to the upregulation of growth factors, such as vascular endothelial growth factor (VEGF), which act on the vascular endothelium, promoting migration and proliferation, and, eventually, the formation of new vessels (140). Angiogenic and inflammatory pathways are also closely associated thus it can be challenging to disassociate the two processes (141).

Recently, several new PET radiotracers have been used to target angiogenesis and repair mechanisms in the myocardium (142). αvβ3 integrin, a transmembrane cell surface receptor that is an important mediator of angiogenesis, and is expressed by macrophages and myofibroblasts after myocardial infarction, is also a target for imaging of angiogenesis using PET (143–145). However, repair processes measured by using integrin αvβ3-targeted radiotracers can be attributed to angiogenesis, fibrosis and inflammation. This is because integrin αvβ3 is not a specific marker of activated endothelial cells, as it is also expressed by macrophages. Despite this limitation, it is frequently reported as a target for quantification of angiogenesis using PET radiotracers (146–149). The αvβ3 integrin also binds to collagen, further complicating the distinction between signals from different targets. Indeed, ^18^F-Fluciclatide, a PET radiotracer used to image αvβ3 expression, has been used to detect fibrosis in the heart after myocardial infarction (41). Other PET radiotracers used for investigating myocardial angiogenesis following infarction include: ^18^F-galacto-RGD (clinical use), ^64^Cu-VEGF, ^18^F-PRGD, ^64^Cu-TRC105, and multiple ^68^Ga-labeled RGD peptides (preclinical use) (Table 2) (42, 43, 148, 150–153). These radiotracers have all shown increased uptake after myocardial infarction at the infarct site. However, targeting with an RGD peptide means the potential labeling of both angiogenesis and inflammation, which can complicate clear interpretation of the results (154).

The nicotinic acetylcholine receptor α7 subtype (α7nAChR) has emerged as an alternative to integrins or VGEF receptors as a target for imaging angiogenesis. This is due to its role in the modulation of important angiogenic signaling pathways (155). Nicotine promotes angiogenesis via the nAChRs, in areas with inflammation, atherosclerosis and ischemia, as well as in areas where tumors are present (156, 157). An *in vitro* study demonstrated that, whilst endothelial cells express several isoforms of the nAChR receptor, the α7nAChR subunit was most highly expressed. Furthermore, inhibition of angiogenesis was only obtained by selectively blocking the α7nAChR subunit (157). Similarly, *in vivo* studies have shown that inhibiting or genetically disrupting the α7nAChRs reduced angiogenesis in response to inflammatory and ischemic stimuli in both the ischemic mouse hind limb and disc angiogenesis models (157). A rat pressure overload model induced by coarctation of the abdominal aorta demonstrated increased α7nAChR protein and mRNA levels in the left ventricle 16 weeks after surgery. Results from that study also showed that the animals developed cardiac hypertrophy and increased microvessel density, with expression of α7nAChR most evident around the blood vessels with degeneration of cardiomyocytes also observed (155). Several radiotracers for these receptors have been used in the context of neuroimaging, the most successful being ^18^F-ASEM, a selective α7nAChR antagonist radiotracer, which is already used in clinical studies for neuropsychiatry (158, 159). ^18^F-NS14490, a new agonist radiotracer for targeting α7nAChR, was first proposed as a potentially useful biomarker in cardiovascular imaging when high uptake was visualized in the brain vasculature of pigs *in vivo* (160).

## Conclusion

Heart failure is an important healthcare issue of rising prevalence, with a large number of cases related to hypertension. There is a chronic and cumulative element of heart failure in terms of disease development and progression that could benefit from early detection to guide patient management via molecular imaging techniques, such as PET imaging. The utility of PET is particularly justified in subtle progressive hypertensive heart disease because it is the clinical imaging technique with highest sensitivity (pM to nM range) for detection of molecular changes *in vivo* and non-invasively. Nonetheless, due to hypertensive heart failure having a less pronounced phenotype compared to for example myocardial infarction or oncological conditions, detection of changes could be more challenging. Currently, pre-clinical PET imaging studies with models of hypertensive heart failure are needed to test the utility of new and emerging selective PET radiotracers with clinical potential in this disease context.

## Author Contributions

VB conducted the literature search for this review article and wrote the first manuscript draft. MM, PH, GG, and AT provided comments and edits to the review paper as well as suggested additional literature for inclusion in this review article. All authors contributed to the article and approved the submitted version.

## Conflict of Interest

The authors declare that the research was conducted in the absence of any commercial or financial relationships that could be construed as a potential conflict of interest.

## Publisher's Note

All claims expressed in this article are solely those of the authors and do not necessarily represent those of their affiliated organizations, or those of the publisher, the editors and the reviewers. Any product that may be evaluated in this article, or claim that may be made by its manufacturer, is not guaranteed or endorsed by the publisher.
